# Controlling the Bioprinting Efficiency of Alginate–Gelatin by Varying Hydroxyapatite Concentrations to Fabricate Bioinks for Bone Tissue Engineering

**DOI:** 10.3390/polym18030314

**Published:** 2026-01-23

**Authors:** Nikos Koutsomarkos, Varvara Platania, Dimitris Vlassopoulos, Maria Chatzinikolaidou

**Affiliations:** 1 Department of Materials Science and Engineering, University of Crete, 70013 Heraklion, Greecevplatania@materials.uoc.gr (V.P.); 2 Institute of Electronic Structure and Laser (IESL), Foundation for Research and Technology Hellas (FORTH), 70013 Heraklion, Greece

**Keywords:** bioprinting, rheology, hydrogel, printability, accuracy, composites

## Abstract

A major objective of this study is to investigate the incorporation of hydroxyapatite nanoparticles (nHA) in a biopolymeric matrix of alginate (Alg) and gelatin (Gel), with particular emphasis understanding how controlled variation in nHA concentration affects rheological, mechanical, printing, and biological performance. Although Alg–Gel blends and nHA-containing hydrogels have been previously explored, a systematic and quantitative correlation between nHA loading, viscoelastic recovery, yield behavior, filament fidelity, and cell viability under optimized bioprinting conditions has not been established. Here, we address this by preparing and evaluating six composite inks (0, 1, 2, 3, 4, and 5% *w*/*v* nHA). The parameters of interest included the printing accuracy, the rheological profile, including over 70% viscosity recovery after 10 s in almost all formulations, the elastic modulus, which was over 10 kPa, and the swelling degree. In addition, pre-osteoblastic cells were embedded in these formulations, subsequently bioprinted, and demonstrated viability over 70% after 7 days. The results advance our understanding on the effect of the chemical composition behind the modification of the properties of the composite materials and their applications for biofabrication. This work contributes quantitative insight into how compositional tuning influences the performance of alginate–gelatin–nHA bioinks for extrusion-based bioprinting applications.

## 1. Introduction

Tissue engineering is a multidisciplinary field that aims to develop therapeutic solutions to repair or replace dysfunctional tissues and organs [1]. Recent strategies to develop polymer-based constructs for bone tissue engineering such as freeze-drying, electrospinning, and gas foaming fail to meet the desired structural characteristics (i.e., porosity, pore size, and pore interconnectivity) and mechanical properties that are critical for the growth of bone tissue [2]. Additive manufacturing technologies have made it possible to tailor bone implants to the individual trauma site and have achieved the fabrication of constructs with ideal mechanical characteristics that approximate those of the native bone [3]. However, all these fabrication techniques restrict the in vitro experimentation solely to cell seeding on top of a scaffold. This approach exhibits a cell distribution problem and provides a limited approximation of the in vivo cellular microenvironment. Bioprinting emerges as a versatile technology that solves this problem by integrating cells with the fabrication process of the construct [4,5].

Bioprinting is a new field that uses a computer-controlled three-dimensional (3D) printing device to accurately deposit cells either alone or mixed with biomaterials (inks) into precise geometries, aiming to create anatomically correct biological structures for tissue engineering and regenerative medicine purposes. Several bioprinting approaches have been recently explored, encompassing the use of extrusion devices, inject-like printers, and laser-assisted devices [6]. The most common printing techniques which are currently being employed are extrusion-based deposition, inkjet bioprinting, stereolithography, and laser-induced forward transfer [7]. In a typical extrusion-based bioprinter, the bioink is loaded into a syringe barrel and is extruded through a micro-nozzle tip either using pneumatic pressure or by mechanical force employing a piston or screw [8,9].

Bioprinted hydrogel blends consisting of alginate (Alg) and gelatin (Gel) have been reported in the literature. Specifically, mouse embryonic stem cells were found to maintain 90% viability after printing and their pluripotency after 7 days in culture [10]. The same research group investigated the rheological characteristics of Alg–Gel hydrogels while proving a correlation between cell viability and holding printing time [11]. Another research group reported on the in vitro formation of tumor co-culture spheroids within the hydrogel and the crosstalk between co-cultured cells [12]. When bioactive glass was added to an alginate–gelatin bioink, human osteosarcoma Saos-2 cells died immediately after printing, but human bone marrow-derived mesenchymal stem cells survived the printing process and maintained good viability for 14 days, suggesting that the viscosity of the bioink affects the short- and long-term viability of cells since it delays diffusion, and therefore the bioink should be tailored to suit the cell type [13]. An overlay of agarose and calcium salt of polyphosphate induced the capacity of Saos-2 cells to proliferate, within an alginate–gelatin scaffold, and when cultured for 7 days in an osteogenic cocktail, the cells reacted strongly with alizarin red stain, suggesting high calcium mineralization [14]. It is evident that Alg–Gel blends exhibit a high impact in tissue engineering by means of bioprinting, particularly in bone tissue engineering, thus we examined their potential as promising candidates for these applications.

Although Alg–Gel blends are widely used in bioprinting, they often lack the mechanical strength, filament stability, and shear recovery required for bone tissue engineering applications. The incorporation of nanohydroxyapatite (nHA) addresses these limitations, as nHA mimics the mineral phase of bone and can reinforce the hydrogel network. However, the effect of nHA concentration on the key parameters governing bioprinting, such as rheology, print fidelity, mechanical properties, and cell viability, has not been systematically evaluated. Understanding how nHA content modulates these properties is essential for the rational design of composite bioinks. Therefore, this study investigates Alg–Gel–nHA inks with controlled nHA concentrations to fabricate biomimetic constructs for bone regeneration. Several composite materials have been explored for bone printing; however, it remains a critical gap to understand the influence of nHA on printing efficacy and cellular response [15]. The focus of this investigation is on the interplay between these parameters and the cell viability investigation.

This study goes beyond prior reports on Alg–Gel-based bioinks by systematically linking compositional tuning (0, 1, 2, 3, 4, and 5% nHA) to quantitative rheological and printing performance metrics under optimized conditions. The integrated evaluation of viscoelastic recovery, printed filament fidelity, and cell viability establishes compositional design tools that can be extrapolated to guide the optimization of other nanocomposite bioinks. Such an integrated evaluation has not been previously reported for Alg–Gel-based nanocomposites. The framework can be adapted to alternative nanofillers or different cell types with distinct shear tolerance, thereby offering a strategy for the rational design of bioprintable composite materials.

## 2. Materials and Methods

### 2.1. Preparation of Alginate–Gelatin–Hydroxyapatite Hydrogels

For the preparation of the Alg–Gel–nHA composite hydrogels, a blend containing final concentrations of 7% *w*/*v* alginate and 8% *w*/*v* gelatin was mixed, and varying amounts of nHA. The nanohydroxyapatite was provided as nanoXIM·Care Paste (Fluidinova, S.A., Matosinhos, Portugal), an aqueous dispersion containing 15% *w*/*v* solid nanohydroxyapatite, as specified by the manufacturer. Transmission electron microscopy data confirmed a rod-like shape of this nHA material with an average length of 20 to 40 nm with a peak at 25 nm, as previously reported in detail [16]. The paste does not involve chemical surface functionalization or added dispersants beyond aqueous stabilization. In this study, nHA concentrations (0–5% *w*/*v*) refer to the final mass of solid nanohydroxyapatite per total hydrogel volume and were calculated based on the known solids content of the nHA paste. The formulations AlgGel-nHA0, AlgGel-nHA1, AlgGel-nHA2, AlgGel-nHA3, AlgGel-nHA4, and AlgGel-nHA5 are designated as presented in Table 1.

The hydroxyapatite paste was first dispersed in either ultrapure water or phosphate-buffered saline (PBS) at pH 7.4 and stirred for 10 min at 70 °C to ensure uniform dispersion. Gelatin (Type B from bovine skin, Sigma-Aldrich, Taufkirchen, Germany) was then added and dissolved for 15 min at 70 °C. Subsequently, alginate (medium-viscosity sodium alginate from brown algae, Sigma-Aldrich, Germany) was incorporated and the mixture was continuously stirred for 2 h until a homogeneous viscous solution was obtained.

The prepared hydrogels were stored at 4 °C until further use. Prior to printing or testing, the hydrogels were gently heated to 37 °C to reach a fluid state. This temperature control step is essential as the gelatin component exhibits thermal hysteresis [16], transitioning from sol to gel state upon cooling below approximately 30 °C and reverting to fluid state when reheated. Maintaining the temperature close to physiological levels ensures consistent viscosity during printing.

### 2.2. Fourier-Transformed Infrared Spectroscopy (FTIR)

FTIR analysis of pure materials and all Alg–Gel–nHA composite blends was performed using an optical spectrometer (Nicolet 6700, Thermo Fisher Scientific, Waltham, MA, USA) equipped with an attenuated total reflectance (ATR) diamond crystal. Spectra were collected in the range of 500–4000 cm^−1^ with a spectral resolution of 4 cm^−1^, averaging 64 scans per sample. Baseline correction and normalization were carried out using the OMNIC software version 9.0. Each sample was measured in triplicate to ensure reproducibility.

### 2.3. Rheological Characterization

All rheological measurements were performed with a rotational rheometer MCR501 (Anton Paar, Graz, Austria), operating in strain-control mode. A stainless steel cone and plate geometry with radius R = 25 mm, cone angle α = 4°, and truncation a = 0.053 mm was used. Prior to testing, each sample was left to equilibrate at room temperature for 1 h. It was then loaded on the rheometer where it was confined with a 5 mPa∙s polydimethylsiloxane oil in order to minimize water evaporation at 37 °C.

Dynamic strain sweep measurements were carried out with shear strain amplitude in the range 0.1–100%, at an angular frequency of 10 rad/s, to determine the linear viscoelastic regime (LVE) (Supplementary Figure S1). Subsequently, dynamic frequency sweep measurements were performed at a strain amplitude of 1% and angular frequency ranging from 100 to 0.01 rad/s. To characterize the material’s viscoelastic recovery, we implemented a protocol comprising a dynamic time sweep at a strain amplitude of 1% and frequency of 1 rad/s for 60 s, followed by a sweep at a strain to 200% for 4 s and, finally, a sweep at the original strain of 1% for 10 min.

To evaluate the recovery’s characteristic time, a non-linear curve was fitted to the data beginning at the final sweep at 1% strain:

y = y_0_ + A_1_ e^(−x/τ)^

where τ is the recovery characteristic time. Finally, transient shear rate tests were performed with a rest time of 1.5 h before the application of a new shear rate, to confirm shear-thinning behavior (Supplementary Figure S2). All rheological measurements were performed in triplicates per formulation (n = 3).

### 2.4. Three-Dimensional Printing Process

#### 2.4.1. Design

**Figure 1:**
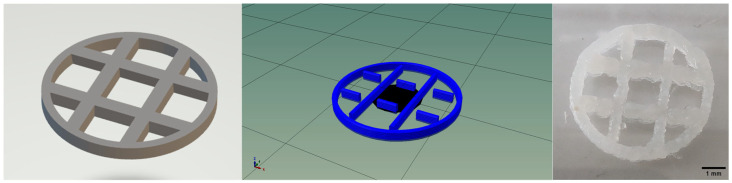
The 3D object as designed with the Tinkercad software (**left**), the sliced and modified g-code model (**middle**), and the 4% nHA result (**right**). Scale bar represents 1 mm.

The design of the multilayered construct was performed using the Tinkercad software (Autodesk, San Francisco, CA, USA, version 2024.2). The 3D object was exported as stl file and processed using Slic3r (version 1.3.0) to generate the corresponding gcode file (Figure 1). The construct has some intersected lines which hindered the multi-layered result. With some modifications in the gcode, the final design was achieved, which was used for all experiments mentioned below.

#### 2.4.2. Printing

Printing was carried out using an extrusion-based bioprinter (Inkredible+ Cellink, Gothenburg, Sweden). The bioink was loaded into a 10 mL syringe connected to a 3 mL cartridge via a female/female Luer-lock adapter and fitted with a 20 G conical nozzle (inner diameter of 0.58 mm). The cartridge was maintained at 37 °C to ensure continuous flow by keeping the gelatin component in sol state, while the printing platform was kept at room temperature (22 ± 1 °C) to promote rapid solidification of the printed filaments upon deposition. A constant pressure of 200 kPa was selected as the optimal across all formulations for the biofabrication of the constructs. Constructs were printed as six-layer grids under optimized conditions (Table 2).

After printing, constructs were immersed in a dual crosslinking solution consisting of 1% (*w*/*v*) CaCl_2_ and 0.025% (*v*/*v*) glutaraldehyde (GA) for 15 min to provide ionic crosslinking of alginate and mild covalent stabilization of gelatin. Since GA is cytotoxic at higher concentrations, to mitigate residual aldehyde and minimize cytotoxicity immediately after crosslinking, constructs were transferred to a 0.1 M glycine solution (in PBS) and incubated for 30 min at room temperature to quench unreacted aldehyde groups. Constructs were then washed with sterile PBS and then incubated in fresh medium at 37 °C. The medium was changed every 3 days. All preparation steps were performed under aseptic conditions. The combined low GA concentration, short exposure time, and the extensive PBS/medium washes were utilized to minimize residual cytotoxic aldehydes while preserving gelatin crosslinking for mechanical stability.

### 2.5. Filament Diameter

**Figure 2:**
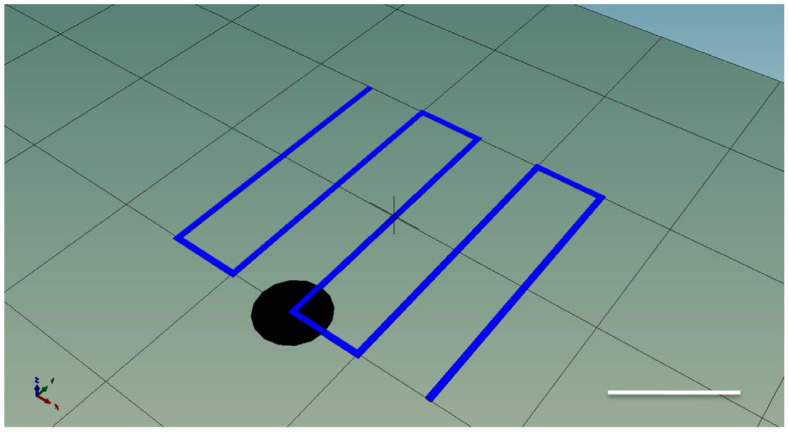
The g-code filament test model. Scale bar represents 10 mm.

To evaluate the fidelity of each bioink formulation, single-layer zig-zag patterns of 20 mm × 20 mm were printed under constant volumetric flow at 0.0088 mL/s and constant pressure of 200 kPa conditions (Figure 2). For experiments conducted under constant volumetric flow conditions, the flow rate was experimentally calibrated for each bioink formulation. At a constant temperature of 37 °C, inks were extruded at different pressures until a steady-state extrusion of 0.5 mL over 57 s was achieved, corresponding to a volumetric flow rate of 0.0088 mL s^−1^. The pressure required to reach this flow rate varied among formulations, reflecting differences in viscosity; however, the volumetric throughput was identical across all inks. Images of the printed filaments were captured using a Leica DM IRE2 (Leica Microsystems, Wetzlar, Germany) inverted microscope equipped with a digital camera. Each image was calibrated, and filament diameter (D_f_) was measured at 40–80 random locations using ImageJ (version 1.54) after automatic thresholding. Printing accuracy was calculated as follows:

Printing accuracy (%) = (D_n_/D_f_) × 100

where D_n_ is the inner diameter of the nozzle (0.58 mm).

Filament diameter measurements were obtained from 40 to 80 locations per construct, from three independently printed constructs per formulation (n = 3).

### 2.6. Mechanical Characterization

The mechanical properties of the crosslinked hydrogels were evaluated under uniaxial compression to determine their bulk elastic modulus. Cylindrical specimens (10 mm in diameter × 20 mm in height) were printed using the same optimized parameters described in Section 2.4 and crosslinked with 1% *w*/*v* CaCl_2_ and 0.025% *v*/*v* glutaraldehyde. The tests were conducted using a mechanical testing system (UniVert, CellScale Biomaterials Testing, Waterloo, ON, Canada) equipped with a 50 N load cell, at room temperature in air. Compression was applied at a constant speed of 15 mm/s. The loading was continued up to 90% strain, and the Young’s modulus was calculated from the slope of the stress–strain curves within the strain range of 5–20% (linear elastic regime). Compression testing was performed in air at room temperature to ensure consistent testing conditions across all samples and to minimize handling time following crosslinking. The reported modulus therefore represents an apparent compressive modulus under these conditions; hydrated testing at lower strain rates may yield different absolute values and was not included in this study. All measurements were performed in 7 ≤ n ≤ 9 replicates, and data are presented as mean ± standard deviation.

### 2.7. Swelling Degree

To measure the swelling degree (Q) of the hydrogels, three constructs were printed as described above and their weight was measured at 0, 1, 3, 6, and 24 h (wet weight). After the measurement, they were freeze-dried for 24 h and measured again (dry weight). The swelling degree was calculated as follows:

Q% = (wet weight − dry weight)/(dry weight) × 100

Measurements were performed on three independently printed constructs per formulation (n = 3).

### 2.8. Bioprinting

#### 2.8.1. Cell Culture

MC3T3-E1 pre-osteoblastic cells (DSMZ, Braunschweig, Germany, ACC-210) were isolated from new-born mouse calvaria [17] and cultured in α-modified minimum essential medium (a-MEM) (PAN-Biotech, Aidenbach, Germany) supplemented with 10% *v*/*v* fetal bovine serum (FBS) (PAN-Biotech, Germany), 100 μg/mL penicillin and streptomycin (PAN-Biotech, Germany), 2 mM L-glutamine (PAN-Biotech, Germany), and 2.5 μg/mL amphotericin (Gibco, Thermo Fisher Scientific, Paisley, UK) in a humidified incubator at 37 °C and 5% CO_2_. The culture medium was replaced twice weekly. The cells were detached using 2.5% *v*/*v* trypsin ethylenediaminetetraacetic acid (EDTA) (Gibco, Thermo Fisher Scientific, UK).

#### 2.8.2. Bioprinting Process

**Figure 3:**
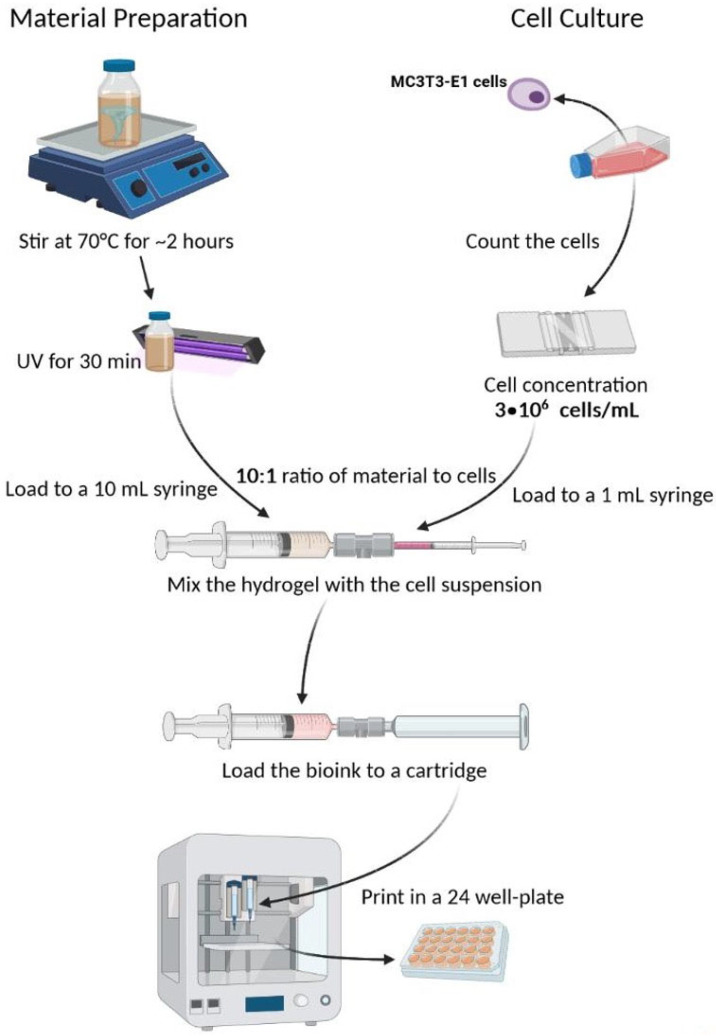
Graphical representation of the ink preparation and the bioprinting process.

For the bioprinting experiments, 90–100% confluent MC3T3-E1 cell culture plates were used. Cells were harvested from near-confluent cultures, detached using trypsin–EDTA, and resuspended at a density of 3 × 10^7^ cells/mL in growth medium. A 1:10 volumetric ratio of cell suspension to hydrogel (100 µL of cell suspension per 900 µL of hydrogel precursor) was used to achieve a final concentration of approximately 3 × 10^6^ cells/mL in the bioink. Mixing was performed through a sterile female/female Luer-lock connector by gentle reciprocal transfer between two syringes to ensure homogeneous cell distribution. Afterward, a similar process was followed as described in Section 2.4.2 with the modification that the printer was moved into the biosafety cabinet to provide aseptic conditions and the bioprinted constructs were washed only with PBS and with cell culture media. Immediately after bioprinting, the constructs were immersed in a solution containing 1% *w*/*v* CaCl_2_ and 0.025% *v*/*v* glutaraldehyde for 15 min to induce dual crosslinking. Calcium ions provided ionic crosslinking of alginate chains, while glutaraldehyde enabled mild covalent crosslinking of gelatin to enhance mechanical stability. After crosslinking, all cell-laden constructs were quenched in 0.1 M glycine in PBS for 30 min to neutralize residual aldehyde groups and thoroughly washed with PBS prior to incubation in culture medium. Constructs were then thoroughly washed with PBS, and transferred to an incubator until further use. Figure 3 displays the steps of the ink preparation and the bioprinting process.

### 2.9. Evaluation of Cell Viability

Cell viability was assessed using a Live/Dead assay (Biotium, Fremont, CA, USA) at days 1 and 7 post-printing. Constructs were stained with 2 µM calcein-AM and 4 µM ethidium homodimer III for 30 min at room temperature, washed with PBS, and imaged on a Leica TCS SP8 confocal microscope (Leica Microsystems, Wetzlar, Germany). For each condition, z-stacks spanning 100 µm in depth were acquired at 1 µm step intervals and subsequently converted to maximum-intensity projections to integrate fluorescence signals across all planes.

Quantification was performed in ImageJ (Fiji distribution). The green (live) and red (dead) channels were thresholded automatically (Otsu’s method), and cells were counted using the “Analyze Particles” plugin. For each construct, three independent z-stacks were analyzed, and the mean viability value was calculated across all projections as

% Viability = N_green_/(N_green_ + N_red_) × 100%

where N_green_: number of cells stained in green, and N_red_: number of cells stained in red. The quantification thus represents the average of three z-stack projections per construct, ensuring a representative measure of total cell viability within the imaged volume.

### 2.10. Statistical Analysis

Statistical analysis was performed using one-way and two-way ANOVA multiple comparison tests in GraphPad Prism software (GraphPad 8.0, San Diego, CA, USA). A *p*-value of <0.05 was considered significant. Data are presented as mean ± standard deviation (SD). For comparisons among multiple groups, one-way ANOVA was applied, followed by Tukey’s multiple comparisons post hoc test. For datasets involving two independent variables (e.g., swelling degree over time), two-way ANOVA was used with Sidak’s multiple comparisons correction. Sample sizes (n) for each experiment are reported in the corresponding Methods subsections and figure captions. A *p*-value of <0.05 was considered statistically significant for all analyses.

## 3. Results

### 3.1. FTIR Characterization

**Figure 4:**
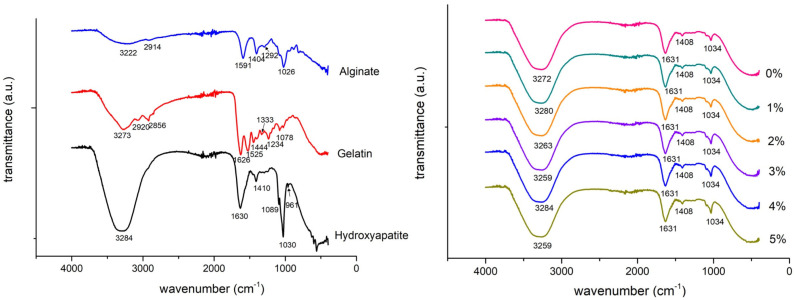
FTIR spectra of pure materials (**left**) and hydrogel blends with various concentrations (0–5%) of hydroxyapatite (**right**).

The FTIR transmittance spectra of the pure materials and all six blends and their peaks are shown in Figure 4. Alginate vibration bands of the CO_2_^−^ were located at 1591 cm^−1^ (antisymmetric stretch) and 1404 cm^−1^ (symmetric stretch). The wide band around 3222 cm^−1^ is due to the stretch of OH and the peak at 2914 cm^−1^ is caused by CH stretching. At 1297 cm^−1^, a skeletal vibration occurs followed by the antisymmetric stretch of C–O–C at 1026 cm^−1^. The spectra of alginate are in agreement with results from Lawrie et al. [17], while gelatin bands from bovine skin were described by Ibrahim et al. [17,18]. The characteristic peaks of amide groups were located at approximately 3273 cm^−1^ due to NH stretching, at 1626 cm^−1^ due to C=O and CN stretching (amide I), at 1525 cm^−1^ (amide II), and at 1234 cm^−1^ (amide III). The vibration of CH_2_ is represented by four bands: at 2920 cm^−1^ (antisymmetric stretch), at 2856 cm^−1^ (symmetric stretch), at 1444 cm^−1^ (bending), and at 1333 cm^−1^ (wagging). The band at 1078 cm^−1^ corresponds to the CH_3_ amide group. Hydroxyapatite showed three characteristic peaks at 961, 1030, and 1089 cm^−1^, which indicate the presence of phosphate ion (PO_4_^3−^) groups. The peak at 1630 and the broad band at 3000–3700 cm^−1^ are attributed to the bands of lattice water. The peak at 1410 cm^−1^ is a result of CO_2_^−^ adsorption by the apatite [16]. All six blends displayed the same bands: a broad band around 3000–3700 cm^−1^ mainly due to water, a peak at 1631 cm^−1^ that is a result of the convergence of water, CO_2_^−^ (antisymmetric), and amide I stretching, a small peak at 1408 cm^−1^ due to CO_2_^−^ (symmetric) stretch, and a peak at 1034 cm^−1^ due to phosphate ion groups.

### 3.2. Rheological Characterization

**Figure 5:**
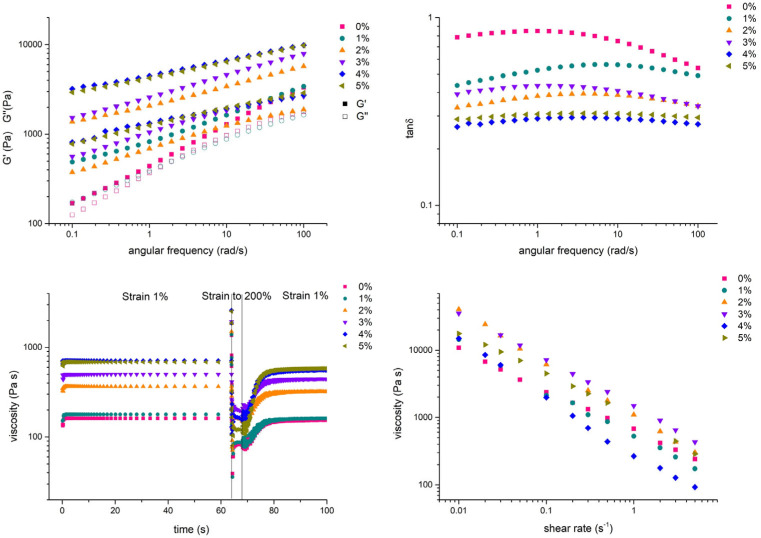
Rheological evaluation of all composites. Storage modulus G′ and loss modulus G″ vs. angular frequency (**upper left**), tangent of the loss angle δ vs. angular frequency (**upper right**), viscosity recovery (**lower left**) and shear-thinning curve (**lower right**).

All ink compositions were rheologically characterized, and the results are presented in Figure 5. The linear viscoelastic data indicate that increasing the concentration of nHA results in a more significant increase in G′ (storage modulus) compared to G″ (loss modulus) values, and a respective decrease in the loss factor (tanδ = G″/G′). Note that the 4% and 5% nHA concentrations behave almost identically. In fact, in the absence of nHA, the native blend exhibits a gel response with moduli nearly collapsed (tanδ nearly frequency-independent), following a power-law dependence on frequency. Finally, the recovery was measured from the rotational recovery measurements. The 0% nHA blend exhibited the highest recovery after 10 s at 81 ± 6% and the lowest was from blend 4 at 66 ± 6%. Table 3 summarizes the recoveries of the blends.

### 3.3. Printing Accuracy

**Figure 6:**
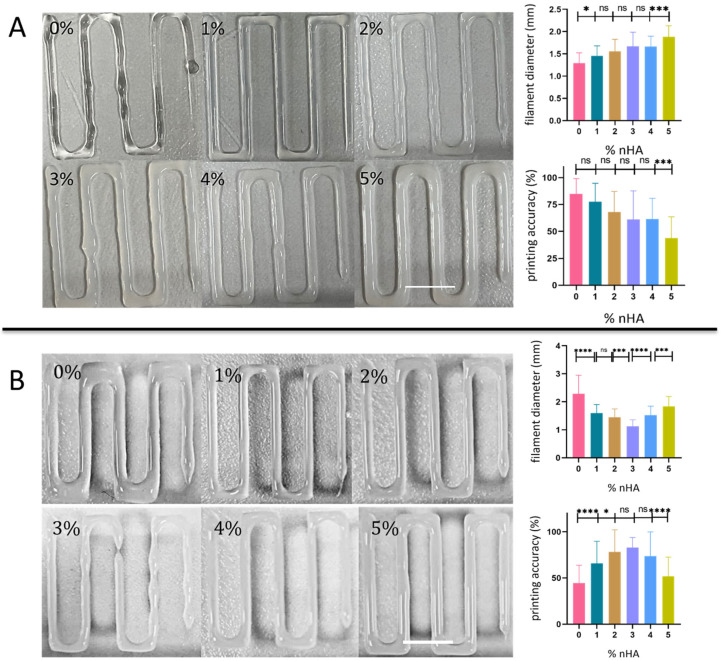
Evaluation of the printability of the bioinks. Representative images of the design pattern used to determine spreading filament diameter, and printing accuracy of all different bioink types. (**A**) Printing under constant volumetric flow of 0.0088 mL/s; (**B**) printing under the same pressure of 200 kPa. Statistical analysis was performed between all composites using one-way ANOVA (* *p* < 0.05, *** *p* < 0.001, **** *p* < 0.0001, ns: non-significant). Error bars denote standard deviation. Scale bars represent 10 mm.

The average filament diameters and the printing accuracy ranging between 50 and 85% are shown in Figure 6 as a function of the nHA concentration. The filament diameter of the constructs that were biofabricated under the same flow rate of 0.0088 mL/s (Figure 6A), diverges from the nozzle inner diameter as the concentration of nHA increases, resulting in non-significantly lower printing accuracy levels between 60 and 80% for the 0–4% samples, with the significantly lower value at below 50% being for the 5% composite. Under the same pressure of 200 kPa (Figure 6B), the augmentation of the nHA content from 0 to 1 and 2% results in a significant increase in filament accuracy above 65%, with the samples 3 and 4% showing 70–80% accuracy being non-significantly different. Only the 5% sample with a 50% accuracy indicates a significantly lower value. These results demonstrate that bioprinting the same material compositions using the same volumetric flow or the same pressure may lead to different fidelity values. This was the case for the 0% sample, which indicated the largest divergence among all samples, possibly due to the lower mechanical strength of the pure hydrogel compared to the composites (1–5% samples) that demonstrated similar fidelity when extruded by the same flow rate or by the same pressure. The low bioprinting fidelity determined for the 0% and the 5% samples may be attributed to their very low and very high viscosity values, respectively, while the highest fidelity of the 3% samples may correlate with their optimal viscosity. Additionally, it should be noted that the use of a needle with a lower inner diameter, while demonstrating more precisely bioprinted filaments with an accuracy between 60 and 93% (Supplementary Figure S3), was correlated with a significant reduction in cell viability, yielding approximately 20% viability at day 8. This result builds upon the fact that the major factor of cell death in extrusion-based bioprinting techniques is the shear stress caused during flow [9,19].

### 3.4. Measurement of the Young’s Modulus

**Figure 7:**
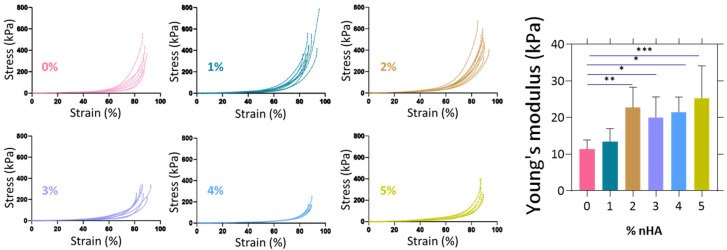
Compressive stress–strain diagrams of the different composite hydrogels and determination of their Young’s modulus values (7 ≤ n ≤ 9 measurements) from 5 to 20% strain regime. Statistical analysis was performed for each composite compared to the control sample 0% nHA, using one-way ANOVA (* *p* < 0.1, ** *p* < 0.01, *** *p* < 0.001). Error bars denote standard deviation.

The elastic modulus of the samples (Figure 7) shows that with increasing concentrations of nHA, the Young’s modulus tends to increase, starting from 11 ± 2 kPa for the 0% to 25 ± 9 kPa for the 5%.

### 3.5. Swelling Degree Analysis

**Figure 8:**
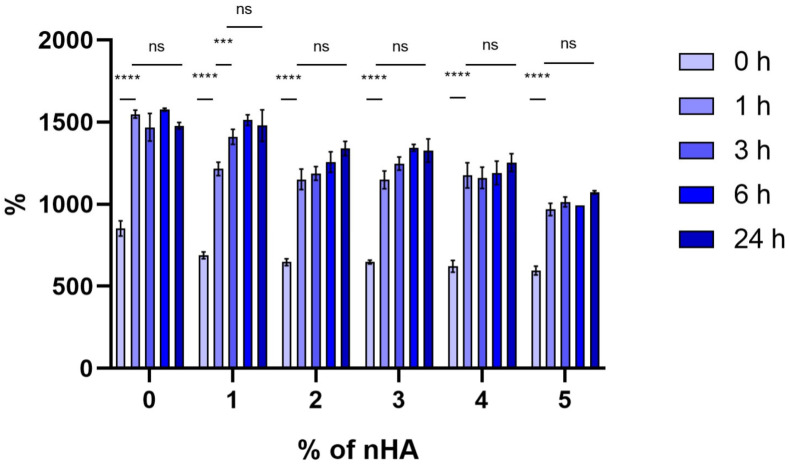
Swelling degree of composite hydrogels measured at 0, 1, 3, 6, and 24 h. Statistical analysis was performed for each composite compared to the previous time point, using two-way ANOVA multiple comparisons (*** *p* < 0.001, **** *p* < 0.0001, ns: non-significant). Error bars denote standard deviation.

Swelling degree analysis (Figure 8) shows that all blends exhibited a significant increase (*p* < 0.0001) in swelling degree between 0 and 1 h and they reached an equilibrium state after 6 h, without significant differences between 3 and 24 h in all blends. Table 4 indicates the % swelling degree at equilibrium.

### 3.6. Cell Viability

**Figure 9:**
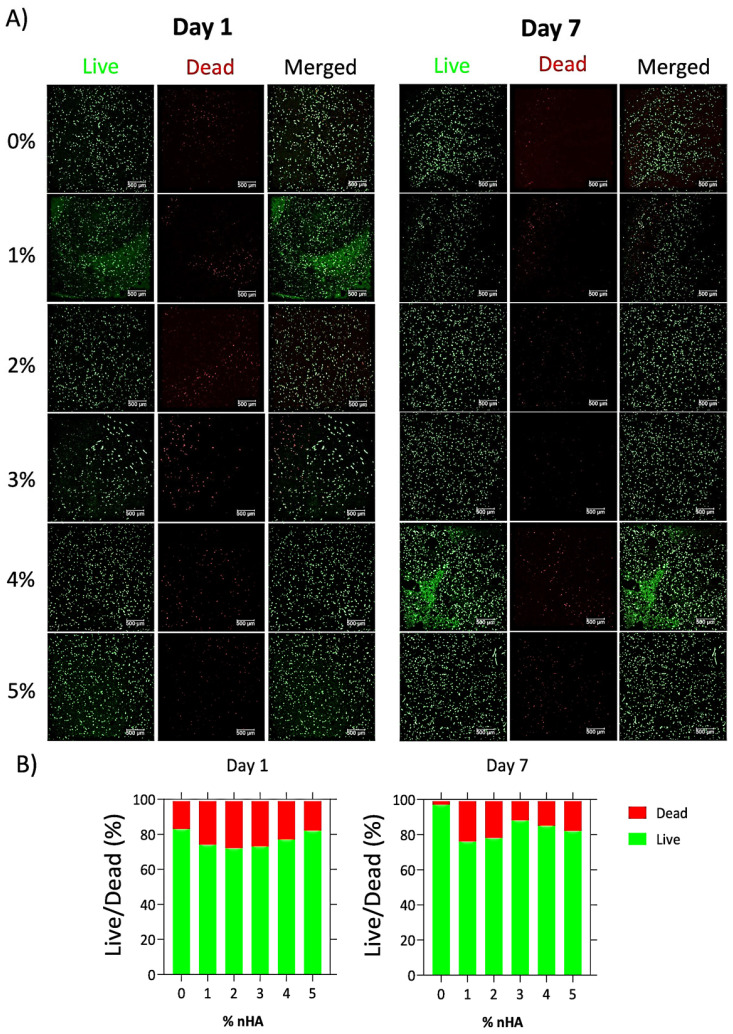
(**A**) Fluorescent staining of pre-osteoblastic cells within the different bioprinted constructs using a Live/Dead assay. Representative confocal microscopy images visualizing in vitro cytocompatibility of the different bioinks. Green fluorescence indicates live cells, and red fluorescence indicates dead cells after 1 (**left column**) and 7 days (**right column**) in culture, scale bar represents 500 μm; (**B**) Quantification of cell viability within the different bioprinted constructs was calculated from the images using Fiji ImageJ software.

Cell viability visualization of the fluorescently labeled cells is presented in Figure 9A, and their quantification is displayed in Figure 9B. On day 1, the cell viability ranged from 72% to 83%. The 0% hydrogel exhibited the highest cell viability at 83%, whereas the 2% composite displayed the lowest viability at 72%. Furthermore, by day 7, the percentage of the viable cells within the bioprinted constructs increased, suggesting an enhanced cell viability inside the composite matrix, except of the 5% counterpart, in which the number of cells between the two time points remained the same. Furthermore, the 0% bioink showed a substantial increase in cell viability, indicating that the Alg–Gel hydrogel is a more favorable environment for cell viability.

## 4. Discussion

The goal of this study was to develop and characterize nanohydroxyapatite (nHA)-reinforced alginate–gelatin (Alg–Gel) hydrogels for use as bioinks in bone tissue engineering. We hypothesized that varying the concentration of nHA would modulate the rheological and mechanical properties of the bioink, thereby influencing its printability and suitability for supporting cell viability. This hypothesis was tested by systematically analyzing six composite formulations (0–5% nHA *w*/*v*) and evaluated their rheological performance, printing fidelity, mechanical behavior, water retention, and pre-osteoblastic cell viability. The results confirm that tuning nHA concentration can be used as a design variable to optimize bioink performance for bone mimetic applications.

Alginate and gelatin are widely used natural biopolymers in bioprinting due to their favorable gelation properties, biocompatibility, and ease of modification. Alginate provides structural support through ionic crosslinking with divalent cations, while gelatin, derived from collagen, offers cell adhesion motifs and thermoresponsive behavior. Together, these components form a composite matrix capable of supporting three-dimensional cell-laden constructs [20]. Incorporation of nHA further aims to mimic bone’s native mineral matrix and encourage osteogenic potential, as demonstrated in recent studies using nHA–polymer bioinks for bone tissue applications [21].

The tangent of the loss angle δ carries information on the ratio between the viscous and the elastic portion of the viscoelastic deformation behavior [22]. In this study, the augmentation of the nHA percentage reduced the tan(δ) of the bioink, which is in accordance with studies reporting that the addition of HA aggravates the viscoelastic behavior of the ink, rendering it more viscous [23,24]. A rheological study on Alg–Gel bioinks focused on printability concluded that when tan(δ) was approximately between 0.25 and 0.45 the Alg–Gel bioinks were printed with relatively good smoothness without compromising structural integrity [25]. The doping of Alg–Gel hydrogels with nHA decreased the recovery of the bioink, which is in agreement with a recent study [23]. This is possibly due to the fact that interactions formed between NH_3_^+^ groups of gelatin with PO_4_^3−^ ions of nHA as well as among the Ca^2+^ ions of nHA and –COO^−^ groups of alginate cease to exist, thus requiring additional time to form back [26,27,28,29].

The rheological testing sequence was designed to progressively evaluate the ink’s deformation behavior under conditions that mimic extrusion bioprinting. Strain sweep tests determined the linear viscoelastic regime (LVE), frequency sweeps characterized viscoelastic dominance (G′ vs. G″) across deformation rates, and time-recovery tests quantified structural reformation after shear. All measurements were performed at 37 °C, and results are summarized in Figure 5 and Table 3. The rheological behavior was then interpreted in the context of printability, filament uniformity, and recovery. The rheological analysis reveals that increasing nHA concentration enhances the elastic component (G′ > G″) while reducing recovery speed, indicating improved structural integrity at the expense of flowability. The optimal printability observed for the 3% nHA formulation corresponds to an intermediate tan δ (0.26–0.43), which balance extrusion continuity and post-deposition stability. Such rheological ranges can serve as quantitative design targets for other composite bioinks where solid reinforcement (e.g., bioactive glass, HA, or nanocellulose particles) can be tuned to maintain printability and cytocompatibility. Beyond the Alg–Gel–nHA system, this structure–property–performance correlation provides a transferable guide for multi-component bioink development. By defining how rheological parameters such as tan δ, τy, and recovery kinetics govern filament shape fidelity and cell survival, the framework may assist in adapting formulations for other tissue engineering contexts or for cell types with varying mechanical sensitivity, including MSCs, chondrocytes, and endothelial cells.

The ability of an ink to extrude as a consistent filament, the formation of the first layer, flow behavior, pressure, and crosslinking capabilities are all properties that significantly impact ink printability [29,30]. It is expected that lower viscosity gels would spread more due to a less dense network, which is evidenced by the lower concentrations exhibiting wider strands [31]. Under the same flow rate, the bioinks with higher concentration of nHA require increasing pressure as their viscosity increases. In this context, the extruded filament becomes less uniform, thereby reducing the printing accuracy [32]. Furthermore, the reduced recovery experienced by the higher concentration of nHA bioinks indicates slower self-healing properties and therefore the ink spreads due to gravitational forces [33]. The doping of nHA in the Alg–Gel matrix increased the accuracy of the bioink until 3% nHA concentration while afterwards it started to reduce. This can be attributed to the fact that the material flow is optimum with the 3% nHA and afterwards the higher viscosity and lower recovery dominate the final result.

Alginate and gelatin hydrogels are typically characterized by elasticity, largely determined by their polymer network, and relatively low mechanical strength, limiting their application in load-bearing environments [34,35]. The observed increase in compressive modulus is due to the incorporation of nHA, which acts as a reinforcement within the polymer matrix. As the nHA content increases, these reinforcing effects become more pronounced, thereby improving the hydrogel ability to resist compressive forces [36,37]. The gradual increase in the compressive modulus from 11 ± 2 kPa to 25 ± 9 kPa as the nHA content increases also suggests that the mechanical properties of the composite hydrogels can be fine-tuned by modulating the nHA concentration. It is important to note that the mechanical properties of the hydrogels are not intended to match those of load-bearing trabecular bone, which exhibits compressive moduli in the MPa range. Instead, the obtained stiffness values of 11–25 kPa fall within the range of unmineralized osteoid and early bone extracellular matrix, supporting their suitability for cell-laden bone tissue models rather than immediate load-bearing applications. The compressive modulus of native cancellous bone extracellular matrix, specifically the unmineralized collagenous osteoid, is reported at approximately 25–40 kPa, while fully mineralized cancellous bone ranges much higher, in the hundreds of MPa to GPa range [38]. Such mechanically compliant environments are suitable for supporting cell encapsulation, osteogenic differentiation, and extracellular matrix deposition, rather than immediate physiological load-bearing applications.

Hydrogels are able to absorb large quantities of water or biological fluids and thus have the potential to be used as matrices for cell growth in tissue engineering [39]. In the present study, the addition of nHA in the Alg–Gel matrix resulted in a decrease in swelling degree as the percentage of nHA increased, a trend confirmed by other reports [40,41,42]. Nevertheless, the degree of swelling degree in all blends was sufficient, and considering the cell viability of this study, the diffusion of nutrients throughout the hydrogel might have been sufficient. MC3T3-E1 pre-osteoblastic cells exhibit, when good attachment occurs, a spindle-like elongated shape [33]. The cell viability images in this study show that the cells have a round-like morphology which could be attributed to the fact that the hydrogel is highly concentrated (15% *w*/*v* in total). In addition, the constituent biopolymers are crosslinked into a dense network; therefore, the cells do not have the necessary space to elongate. Hence, the cells seem to be constricted within the hydrogel matrix, and they possibly experience mechanical stresses. The material concentrations in the ink play a critical role in cell behavior, as cells encapsulated inside a stiff biopolymer matrix present a limited capacity for spreading and proliferating [43,44], as exhibited by the 5% composite construct. On the other hand, cells exhibited high viability (>70%) both at day 1 and day 7, indicating the suitability of the bioinks for bone tissue engineering. Other research studies report on the critical interplay between the printing accuracy and the cell viability within the ink network. Another report on the evaluation of gelatin/alginate bioinks demonstrated an exponential decline in embryonic stem cell viability with increased induced shear stress, despite improved filament fidelity at higher viscosities and printing pressures [11]. Similarly, in vessel-like constructs, increased alginate concentration and smaller coaxial nozzle size (e.g., 23G vs. 26G) led to a sharp drop in initial cell viability from ~89% to ~36%, even though higher filament accuracy was achieved [45]. These findings align with our observations, where a smaller nozzle diameter of 200 µm delivered finer strands but resulted in viability as low as 20% by day 8, ultimately leading us to adopt a larger nozzle diameter of 580 µm to better preserve cell survival. The development of advanced nozzle designs (e.g., shear-optimized geometry or dynamically actuated heads) could allow finer resolution without compromising cell integrity. Moreover, investigating photo-crosslinkable and enzymatically degradable fillers could enhance both structural stability and biological remodeling capabilities. Finally, long-term in vitro and in vivo studies examining osteogenic markers and vascularization within printed constructs would be essential to validate the translational potential of Alg–Gel–nHA bioinks.

## 5. Conclusions

This study demonstrates that controlled incorporation of nanohydroxyapatite is an effective strategy for tuning the rheological behavior, print fidelity, and mechanical performance of Alg–Gel bioinks. By systematically correlating nHA concentration with viscoelastic recovery, filament fidelity, mechanical stiffness, and cell viability, this work establishes quantitative design criteria for extrusion-based bioprinting of bone-mimetic hydrogels. All formulations supported short-term viability of pre-osteoblastic cells at values >70%, confirming their cytocompatibility under optimized printing conditions. An intermediate nHA concentration at 3% *w*/*v* provided the most favorable balance between print accuracy, structural stability, and cellular response, highlighting its near-term potential for fabricating cell-laden bone tissue models. While the present study focused on material and printing performance, future investigations may focus on long-term osteogenic differentiation, matrix mineralization, and in vivo validation. Overall, this work provides a transferable framework for the rational design of nanocomposite bioinks and supports their use in bone tissue engineering and advanced biofabrication applications.

## Figures and Tables

**Table 1:** Name designation of each ink formulation based on their chemical composition.

Name	Composition
0%	7% *w*/*v* Alg and 8% *w*/*v* Gel
1%	7% *w*/*v* Alg, 8% *w*/*v* Gel and 1% *w*/*v* nHA
2%	7% *w*/*v* Alg, 8% *w*/*v* Gel and 2% *w*/*v* nHA
3%	7% *w*/*v* Alg, 8% *w*/*v* Gel and 3% *w*/*v* nHA
4%	7% *w*/*v* Alg, 8% *w*/*v* Gel and 4% *w*/*v* nHA
5%	7% *w*/*v* Alg, 8% *w*/*v* Gel and 5% *w*/*v* nHA

**Table 2:** Printing conditions.

Temperature (°C)	Nozzle Inner Diameter (mm)	Printing Speed (mm/s)
37	0.58	20

**Table 3:** Blend recovery at 10 s and recovery characteristic time.

Recovery at 10 s (%)	τ (s)	Concentration of nHA (%)
81 ± 6	6.2 ± 0.2	0
76 ± 6	6.6 ± 0.2	1
76 ± 5	5.8 ± 0.1	2
76 ± 6	5.7 ± 0.1	3
66 ± 6	5.7 ± 0.1	4
71 ± 7	5.7 ± 0.1	5

**Table 4:** Swelling degree (Q) at equilibrium.

Percentage of nHA (%)	Q at Equilibrium (%)
0	1575 ± 9
1	1510 ± 30
2	1260 ± 60
3	1340 ± 20
4	1190 ± 70
5	994 ± 3

## Data Availability

The original contributions presented in this study are included in the article/Supplementary Materials. Further inquiries can be directed to the corresponding authors.

## Supplementary Materials

The following supporting information can be downloaded at: https://www.mdpi.com/article/10.3390/polym18030314/s1, Figure S1: Determination of linear viscoelastic regime (LVE) from dynamic strain sweep test of 0% to 5% nHA. All compositions showed elastic behavior until 1% shear strain which was used in later experiments. Full shapes denote storage modulus G′ and hollow shapes, loss modulus G″; Figure S2: Transient shear rate tests for various shear rates (ranging from 0.01 s^−1^ to 5 s^−1^) for all material compositions including 0 to 5 *w*/*v*% nHA. The shear rate was estimated at the plateau where the viscosity reached a steady state; Figure S3: Evaluation of the printability of the bioinks. Representative images of the design pattern used to determine spreading filament diameter and printing accuracy of all different composite bioinks using a 22 G (0.41 mm inner diameter) needle for the same weight of extruded filament 0.02 g. Statistical analysis was performed for each composite compared to the previous sample, using one-way ANOVA (* *p* < 0.1, *** *p* < 0.001). Error bars denote standard deviation.

## References

[1] Chandra P.K., Soker S., Atala A., Lanza R., Langer R., Vacanti J.P., Atala A. (2020). Chapter 1—Tissue engineering: Current status and future perspectives. Principles of Tissue Engineering.

[2] Collins M.N., Ren G., Young K., Pina S., Reis R.L., Oliveira J.M. (2021). Scaffold Fabrication Technologies and Structure/Function Properties in Bone Tissue Engineering. Adv. Funct. Mater..

[3] Pawelec K.M., Planell J.A. (2019). Bone Repair Biomaterials: Regeneration and Clinical Applications.

[4] Zhang L.G., Fisher J.P., Leong K.W. (2015). 3D Bioprinting and Nanotechnology in Tissue Engineering and Regenerative Medicine.

[5] Loukelis K., Koutsomarkos N., Mikos A.G., Chatzinikolaidou M. (2024). Advances in 3D bioprinting for regenerative medicine applications. Regen. Biomater..

[6] Atala A., Yoo J.J. (2015). Essentials of 3D Biofabrication and Translation.

[7] Khademhosseini A., Camci-Unal G. (2018). 3D Bioprinting in Regenerative Engineering: Principles and Applications.

[8] Harley W.S., Li C.C., Toombs J.T., O’Connell C.D., Taylor H.K., Heath D.E., Collins D.J. (2021). Advances in biofabrication techniques towards functional bioprinted heterogeneous engineered tissues: A comprehensive review. Bioprinting.

[9] Boularaoui S., Al Hussein G., Khan K.A., Christoforou N., Stefanini C. (2020). An overview of extrusion-based bioprinting with a focus on induced shear stress and its effect on cell viability. Bioprinting.

[10] Ouyang L., Yao R., Mao S., Chen X., Na J., Sun W. (2015). Three-dimensional bioprinting of embryonic stem cells directs highly uniform embryoid body formation. Biofabrication.

[11] Ouyang L., Yao R., Zhao Y., Sun W. (2016). Effect of bioink properties on printability and cell viability for 3D bioplotting of embryonic stem cells. Biofabrication.

[12] Mondal A., Gebeyehu A., Miranda M., Bahadur D., Patel N., Ramakrishnan S., Rishi A.K., Singh M. (2019). Characterization and printability of Sodium alginate -Gelatin hydrogel for bioprinting NSCLC co-culture. Sci. Rep..

[13] Ojansivu M., Rashad A., Ahlinder A., Massera J., Mishra A., Syverud K., Finne-Wistrand A., Miettinen S., Mustafa K. (2019). Wood-based nanocellulose and bioactive glass modified gelatin-alginate bioinks for 3D bioprinting of bone cells. Biofabrication.

[14] Neufurth M., Wang X., Schröder H.C., Feng Q., Diehl-Seifert B., Ziebart T., Steffen R., Wang S., Müller W.E.G. (2014). Engineering a morphogenetically active hydrogel for bioprinting of bioartificial tissue derived from human osteoblast-like SaOS-2 cells. Biomaterials.

[15] Loukelis K., Helal Z.A., Mikos A.G., Chatzinikolaidou M. (2023). Nanocomposite Bioprinting for Tissue Engineering Applications. Gels.

[16] Kavasi R.M., Coelho C.C., Platania V., Quadros P.A., Chatzinikolaidou M. (2021). In Vitro Biocompatibility Assessment of Nano-Hydroxyapatite. Nanomaterials.

[17] Lawrie G., Keen I., Drew B., Chandler-Temple A., Rintoul L., Fredericks P., Grøndahl L. (2007). Interactions between Alginate and Chitosan Biopolymers Characterized Using FTIR and XPS. Biomacromolecules.

[18] Ibrahim M., Mahmoud A.A., Osman O., Abd el-Aal M., Eid M. (2011). Molecular spectroscopic analyses of gelatin. Spectrochim. Acta A Mol. Biomol. Spectrosc..

[19] Paxton N., Smolan W., Böck T., Melchels F., Groll J., Jungst T. (2017). Proposal to assess printability of bioinks for extrusion-based bioprinting and evaluation of rheological properties governing bioprintability. Biofabrication.

[20] Chen X.B., Fazel Anvari-Yazdi A., Duan X., Zimmerling A., Gharraei R., Sharma N.K., Sweilem S., Ning L. (2023). Biomaterials / bioinks and extrusion bioprinting. Bioact. Mater..

[21] Loukelis K., Kontogianni G.I., Vlassopoulos D., Chatzinikolaidou M. (2025). Extrusion-Based 3D Bioprinted Gellan Gum/Poly(vinyl alcohol)/Nano-Hydroxyapatite Composite Bioinks Promote Bone Regeneration. Adv. Heal. Mater..

[22] Petta D., Grijpma D.W., Alini M., Eglin D., D’Este M. (2018). Three-Dimensional Printing of a Tyramine Hyaluronan Derivative with Double Gelation Mechanism for Independent Tuning of Shear Thinning and Postprinting Curing. ACS Biomater. Sci. Eng..

[23] Adhikari J., Perwez M.S., Das A., Saha P. (2021). Development of hydroxyapatite reinforced alginate–chitosan based printable biomaterial-ink. Nano-Struct. Nano-Objects.

[24] Wüst S., Godla M.E., Müller R., Hofmann S. (2014). Tunable hydrogel composite with two-step processing in combination with innovative hardware upgrade for cell-based three-dimensional bioprinting. Acta Biomater..

[25] Gao T., Gillispie G.J., Copus J.S., Pr A.K., Seol Y.J., Atala A., Yoo J.J., Lee S.J. (2018). Optimization of gelatin-alginate composite bioink printability using rheological parameters: A systematic approach. Biofabrication.

[26] Diloksumpan P., de Ruijter M., Castilho M., Gbureck U., Vermonden T., van Weeren P.R., Malda J., Levato R. (2020). Combining multi-scale 3D printing technologies to engineer reinforced hydrogel-ceramic interfaces. Biofabrication.

[27] Sharma C., Dinda A.K., Potdar P.D., Chou C.F., Mishra N.C. (2016). Fabrication and characterization of novel nano-biocomposite scaffold of chitosan-gelatin-alginate-hydroxyapatite for bone tissue engineering. Mater. Sci. Eng. C Mater. Biol. Appl..

[28] Jungst T., Smolan W., Schacht K., Scheibel T., Groll J. (2016). Strategies and Molecular Design Criteria for 3D Printable Hydrogels. Chem. Rev..

[29] Zhang Z., Jin Y., Yin J., Xu C., Xiong R., Christensen K., Ringeisen B.R., Chrisey D.B., Huang Y. (2018). Evaluation of bioink printability for bioprinting applications. Appl. Phys. Rev..

[30] Klotz B.J., Gawlitta D., Rosenberg A., Malda J., Melchels F.P.W. (2016). Gelatin-Methacryloyl Hydrogels: Towards Biofabrication-Based Tissue Repair. Trends Biotechnol..

[31] Di Giuseppe M., Law N., Webb B., Macrae R.A., Liew L.J., Sercombe T.B., Dilley R.J., Doyle B.J. (2018). Mechanical behaviour of alginate-gelatin hydrogels for 3D bioprinting. J. Mech. Behav. Biomed. Mater..

[32] Gillispie G., Prim P., Copus J., Fisher J., Mikos A.G., Yoo J.J., Atala A., Lee S.J. (2020). Assessment methodologies for extrusion-based bioink printability. Biofabrication.

[33] Schwab A., Levato R., D’Este M., Piluso S., Eglin D., Malda J. (2020). Printability and Shape Fidelity of Bioinks in 3D Bioprinting. Chem. Rev..

[34] Ghezzi B., Foresti R., Scialoia L.P., Botti M., Mersanne A., Ratto F., Rossi F., Martini C., Perini P., Favari E. (2025). Preliminary Evaluation of 3D-Printed Alginate/Gelatin Scaffolds for Protein Fast Release as Suitable Devices for Personalized Medicine. Biomedicines.

[35] Shan Y., Li C., Wu Y., Li Q., Liao J. (2019). Hybrid cellulose nanocrystal/alginate/gelatin scaffold with improved mechanical properties and guided wound healing. RSC Adv..

[36] Sánchez-Fernández J.A., Presbítero-Espinosa G., Peña-Parás L., Pizaña E.I.R., Galván K.P.V., Vopálenský M., Kumpová I., Elizalde-Herrera L.E. (2021). Characterization of Sodium Alginate Hydrogels Reinforced with Nanoparticles of Hydroxyapatite for Biomedical Applications. Polymers.

[37] Suvarnapathaki S., Wu X., Lantigua D., Nguyen M.A., Camci-Unal G. (2020). Hydroxyapatite-Incorporated Composite Gels Improve Mechanical Properties and Bioactivity of Bone Scaffolds. Macromol. Biosci..

[38] Kanungo B.P., Gibson L.J. (2010). Density-property relationships in collagen-glycosaminoglycan scaffolds. Acta Biomater..

[39] Chai Q., Jiao Y., Yu X. (2017). Hydrogels for Biomedical Applications: Their Characteristics and the Mechanisms behind Them. Gels.

[40] Wenz A., Borchers K., Tovar G.E.M., Kluger P.J. (2017). Bone matrix production in hydroxyapatite-modified hydrogels suitable for bone bioprinting. Biofabrication.

[41] Zuo Y., Liu X., Wei D., Sun J., Xiao W., Zhao H., Guo L., Wei Q., Fan H., Zhang X. (2015). Photo-cross-linkable methacrylated gelatin and hydroxyapatite hybrid hydrogel for modularly engineering biomimetic osteon. ACS Appl. Mater. Interfaces.

[42] Zhou X., Zhu W., Nowicki M., Miao S., Cui H., Holmes B., Glazer R.I., Zhang L.G. (2016). 3D Bioprinting a Cell-Laden Bone Matrix for Breast Cancer Metastasis Study. ACS Appl. Mater. Interfaces.

[43] Ostrovidov S., Salehi S., Costantini M., Suthiwanich K., Ebrahimi M., Sadeghian R.B., Fujie T., Shi X., Cannata S., Gargioli C. (2019). 3D Bioprinting in Skeletal Muscle Tissue Engineering. Small.

[44] Stepanovska J., Supova M., Hanzalek K., Broz A., Matejka R. (2021). Collagen Bioinks for Bioprinting: A Systematic Review of Hydrogel Properties, Bioprinting Parameters, Protocols, and Bioprinted Structure Characteristics. Biomedicines.

[45] Yu Y., Zhang Y., Martin J.A., Ozbolat I.T. (2013). Evaluation of cell viability and functionality in vessel-like bioprintable cell-laden tubular channels. J. Biomech. Eng..

