# Supplementary material for: Controlling the Bioprinting Efficiency of Alginate–Gelatin by Varying Hydroxyapatite Concentrations to Fabricate Bioinks for Bone Tissue Engineering
Source: Polymers (Basel). 2026 Jan 23;18(3):314. doi: 10.3390/polym18030314 (PMC12899812; doi:10.3390/polym18030314)
Supplement: Supplementary file 1 [file polymers-18-00314-s001.zip › polymers-4066250-supplementary.pdf]

SUPPLEMENTARY

Controlling the bioprinting efficiency of alginate-gelatin by varying hydroxyapatite concentrations to fabricate bioinks for bone tissue engineering

Nikos Koutsomarkos[1#], Varvara Platania[1#], Dimitris Vlassopoulos[1,2], Maria Chatzinikolaidou[1,2,*]

[1] Department of Materials Science and Engineering, University of Crete, Heraklion, Greece

[2] Foundation for Research and Technology Hellas (FORTH)-IESL, Heraklion, Greece

* mchatzin@materials.uoc.gr

[#] equally contributing authors

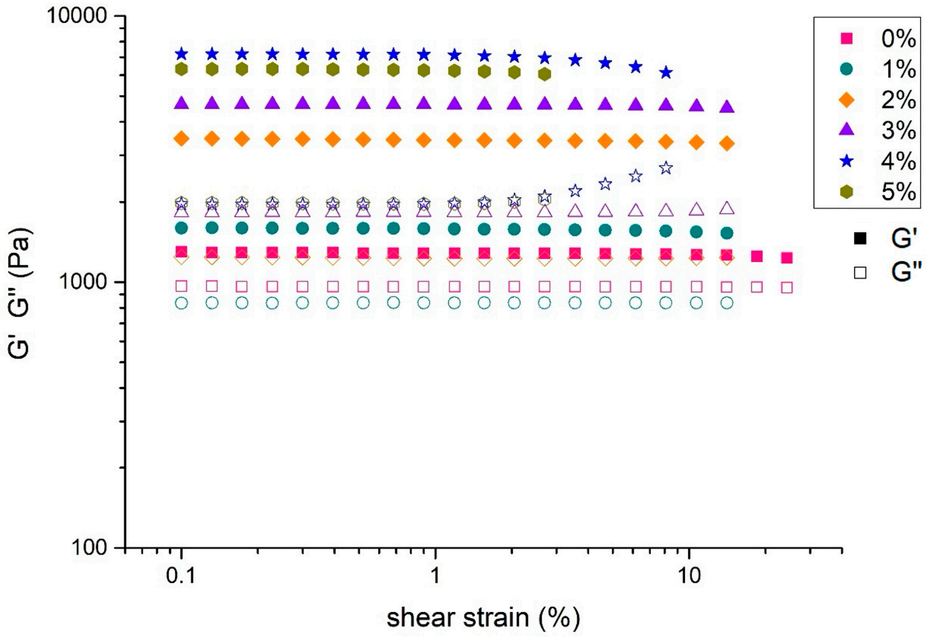

Supplementary Figure S1. Determination of linear viscoelastic regime (LVE) from dynamic strain sweep test of 0% to 5% nHA. All compositions showed elastic behavior until 1% shear strain which was used in later experiments. Full shapes denote storage modulus G′ and hollow shapes, loss modulus G′′.

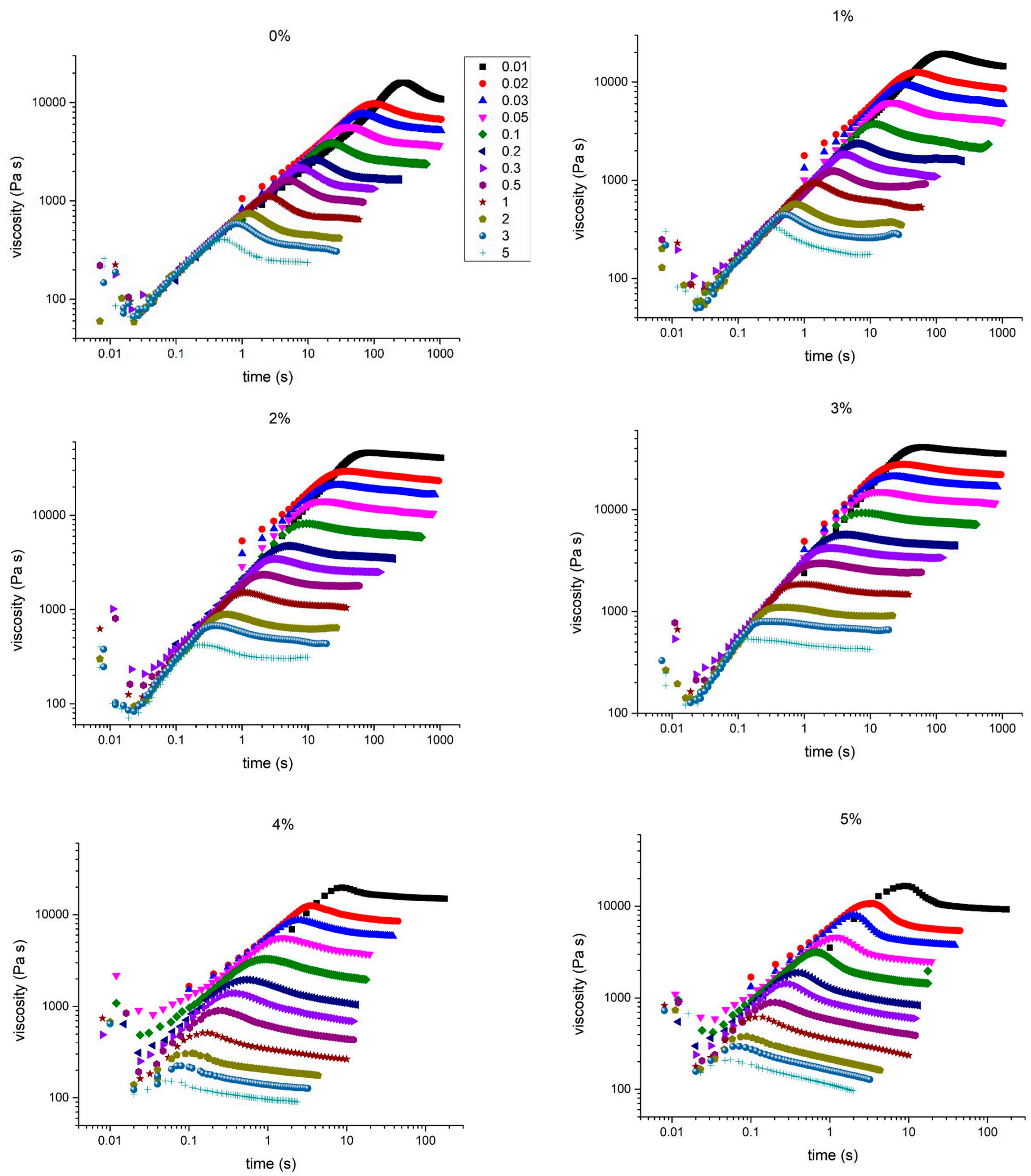

Supplementary Figure S2. Transient shear rate tests for various shear rates (ranging from 0.01 s^{-1} to 5 s^{-1}) for all material compositions including 0 to 5 w/v% nHA. The shear rate was estimated at the plateau where the viscosity reached a steady state.

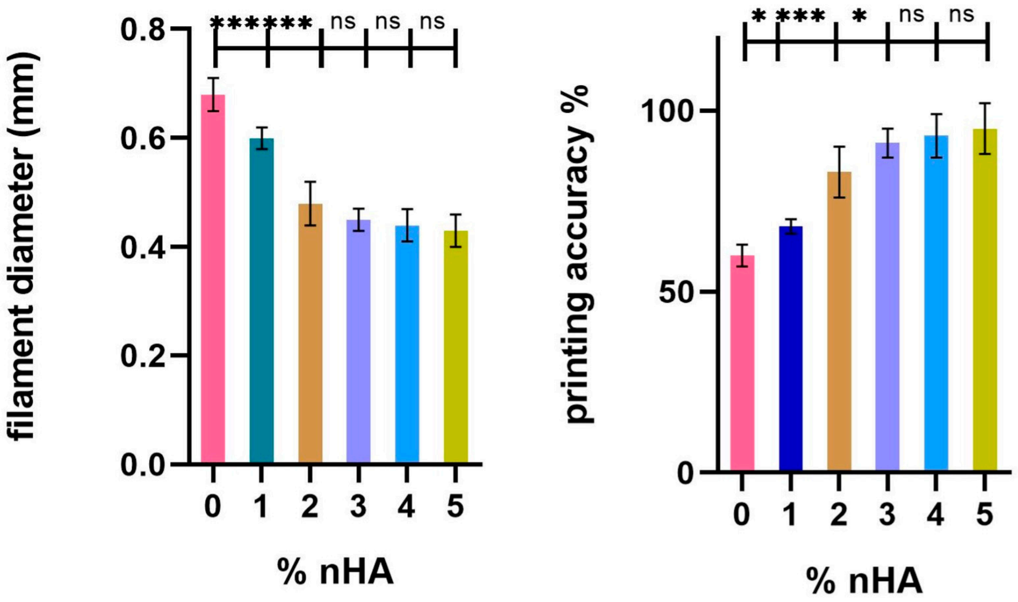

Supplementary Figure S3. Evaluation of the printability of the bioinks. Representative images of the design pattern used to determine spreading filament diameter and printing accuracy of all different composite bioinks using a 22G (0.41 mm inner diameter) needle for the same weight of extruded filament 0.02 g. Statistical analysis was performed for each composite compared to the previous sample, using one-way ANOVA (* $p < 0.1$, *** $p < 0.001$). Error bars denote standard deviation.
